# Mechanical function of the unique alveolar torus in the sabretooth *Nimravus brachyops* (Nimravidae, Carnivora)

**DOI:** 10.1098/rsbl.2025.0208

**Published:** 2025-08-27

**Authors:** Z. Jack Tseng, Narimane Chatar

**Affiliations:** ^1^ Department of Integrative Biology, University of California, Berkeley, CA, USA

**Keywords:** sabretooth, alveolar torus, mandible

## Abstract

Sabretoothed mammals exemplify some of the most extreme craniodental morphological specializations in vertebrates. Much attention has been devoted to their elongated upper canines; however, not all sabretooths possess the same complex of morphological characteristics associated with sabres, making generalization of the requirements for specialized jaw function difficult. Here, we test the approximately 150-year-old hypothesis that a unique jaw torus seen in a single sabretooth genus, *Nimravus*, is an adaptation to resist biting forces. We tested a suite of biting scenarios using finite element analysis and found that the inclusion of a torus structure decreased the performance of the mandible in its stiffness and strain resistance but increased simulated bite force as well as efficiency. The presence of a torus also preferentially improved the overall performance of the mandible at higher gape angles, configurations often inferred for sabretooths. Lastly, a potential novel torus-associated portion of the masseter muscle would have further increased bite performance. The strong association between morphology and performance suggests that the torus may have played a mechanical role in mastication, and its apparent unique evolution is another prime example of mosaic evolution in the sabretooth functional morphology.

## 1. Introduction

**Figure 1.**
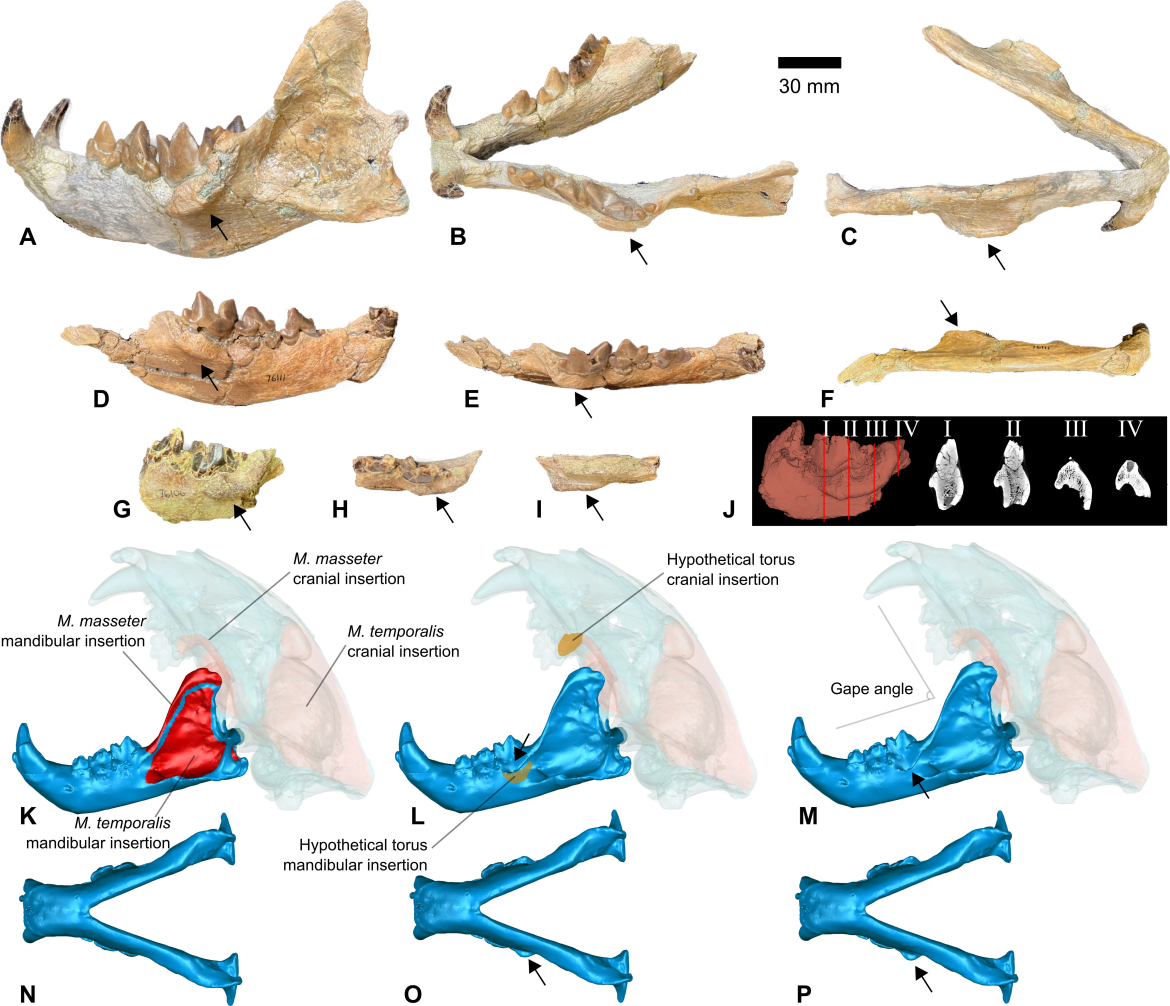
Examples of alveolar tori in *Nimravus brachyops* specimens and model parameters for jaw biting simulations using *Panthera onca*. (A) *N. brachyops* mandible, UCMP (University of California Museum of Palaeontology) 76603, lateral view, (B) UCMP 76603, occlusal view, (C) UCMP 76603, ventral view, (D) *N. brachyops* right partial dentary, UCMP 76111, lateral view. (E) UCMP 76111, occlusal view. (F) UCMP 76111, ventral view. (G) *N. brachyops* left denary fragment, UCMP 76106, lateral view. (H) UCMP 76106, occlusal view. (I) UCMP 76106, ventral view. (J) Alveolar torus cross-sectional views of UCMP 76106, (K) *P. onca,* MVZ (Museum of Vertebrate Zoology, University of California, Berkeley) 4900, lateral skull model view. (L) Small torus model, lateral view. (M) Large torus model, lateral view. (N) MVZ 4900 ventral view of mandible. (O) Small torus model, ventral view of mandible. (P) Large torus model, ventral view of mandible. Arrows indicate the positions of alveolar tori. The scale bar is for parts A–I only.

Sabretooths are morphologically united by the presence of elongate canine teeth, but are otherwise phylogenetically and phenotypically diverse as an extinct ecological morphology. Recent work on sabretooth functional morphology has revealed evidence of a functional continuum in jaw mechanics [1], optimality in sabre shape [2], heterochronic timing of canine eruption to maintain canine bending strength [3] and even functional convergence with non-sabretooth taxa [4]. The emerging picture from these studies is one of a highly mosaic mode of evolution in multiple sabretooth lineages, with some traits (e.g. reduced coronoid process) occurring in most or all terminal sabretooth taxa and others (e.g. hypertrophied mandibular flange) occurring in only a few taxa. Among the rarest of the morphological traits associated with a sabretooth taxon is the presence of a mandibular alveolar torus, which is a lateral bone growth on the labial side of the mandibular corpus at the position immediately ventral to the first molar (carnassial) tooth (figure 1A–I; electronic supplementary material, figure S1).

The alveolar torus is observed in only a single taxon of sabretooths, the namesake genus of the family Nimravidae, *Nimravus* [5]. *Nimravus* is known from the Eocene to ?Miocene deposits of the Holarctic region, with western North American occurrences exhibiting the most developed alveolar tori. However, as early as 1872, Filhol [6] reported a strong alveolar torus in some *Nimravus intermedius* specimens from the Quercy deposits in France. Piveteau [7] confirmed the consistent presence of this structure in both North American and European *Nimravus* specimens and rejected a pathological origin, noting its uniformity and potential association with masticatory muscles. The functional significance of the torus has long been debated. As early as 1879, E.D. Cope stated that the alveolar torus ‘is evidently a provision against the weakness of the mandibular rami, at the point of greatest strain’ [8, p. 372] and in 1910 Matthew added that the torus was ‘designed apparently to shift the support of the lower carnassial outwards in such a way as to afford space for a more powerful masseter attachment than the construction of the skull and jaws would otherwise admit’ [9]. Subsequently, Toohey [10] formally named the structure and undertook the first histological analysis of the structure, which revealed normal bone architecture and led him to reject the hypothesis of pathology (electronic supplementary material, figure S1).

After a comprehensive review of the functional implications of the mandibular torus, Toohey concluded that no definitive function could be assigned to the torus [10]. However, he observed a possible correlation between torus development, individual age and stratigraphic age, with larger tori occurring in more mature individuals and/or in geologically younger specimens. This correlation was later challenged by Peigné [11], who found no consistent relationship between the age of the individual and torus development, though he did confirm that the structure was more frequently present in specimens from the John Day Formation (in Oregon, USA) than in those further east, from the Great Plains. As it stands, the only functional interpretations that remain under consideration are mechanical reinforcement in the area of greatest strain in the mandible or accommodation for muscular insertion [8,9]. Yet, both those alternative interpretations have been proposed without any supporting biomechanical or anatomical analysis. Besides several additional mentions by other researchers doing taxonomic research on the taxon, no new data have been brought to bear on the question of the potential function of the unique structure.

We use digital modelling on an extant model system to test the hypotheses that (i) an alveolar torus decreases the strain on the mandible during biting and (ii) a potential novel muscle attachment at the alveolar torus increases simulated bite force magnitude and efficiency. This study represents the first attempt in nearly 150 years since the first published observation of the alveolar torus in *Nimravus* to quantitatively test the potential functional role of the osteological feature in the performance of the masticatory system.

## 2. Material and methods

We used an extant felid, *Panthera onca* (Museum of Vertebrate Zoology, University of California, Berkeley, specimen MVZ 4900), as a reference specimen on which to introduce alveolar tori. We chose an extant felid model rather than using *Nimravus* fossils because we wished to minimize the potential influence of deformation and incomplete preservation of fossils on simulation results; furthermore, the introduction of tori onto a torus-less model allowed us to more directly test mechanical performance differences along a theoretical torus absence to presence spectrum, rather than having to determine the exact extent of the torus on a *Nimravus* model and be able to completely reduce/remove the structure during modelling. Pantherin felids are medium to large-sized cats with superficial resemblance to the skull morphology of extinct cat-like carnivorans such as nimravids. The specimen is a complete skull (cranium and mandible) of an adult individual, exhibiting some degree of asymmetry in the tooth wear and mandibular corpus shape (figure 1). The cranium and mandible were CT-scanned separately using a GE Phoenix Nanotom M in the Functional Anatomy and Vertebrate Evolution Laboratory, University of California, Berkeley. A voltage of 100 kV and a current of 150 μA were used; 2500 project images were collected to reconstruct image stacks with an isotropic voxel size of 66.63 μm. This resulted in 3883 images for the cranium and 2829 images for the mandible.

Each CT image stack was imported into 3D Slicer (https://www.slicer.org/) using the stack input/output module of the SlicerMorph library [12]. Given the clear contrast between the bone specimen and the surrounding voxels representing air and a thin plastic holder used to stabilize the specimen during scanning, we used the Otsu automatic thresholding algorithm implemented in the segment editor module in 3D Slicer to select the bone for three-dimensional meshing. We then grew and shrunk the entire segmentation by three voxels to clean up small internal bony processes that interfere with the finite element meshing process. This operation also ensured that small voxels representing thin trabeculae are removed from the model, so the homogeneous material property protocol we applied to the jaw models (see below) is not overly stiffened by the presence of extensive trabeculae with cortical bone properties. The segmentations were converted to three-dimensional meshes with default 0.5 smoothing and exported from 3D Slicer as .stl files.

The stl mesh models were then imported into Geomagic Wrap (Hexagon AB, Stockholm, Sweden). The mandible mesh was decimated to approximately 300 000 triangles while enforcing a maximum aspect ratio of 10 to remove highly skewed triangular elements. Decimation was done sequentially in 20% increments from an initial model size of approximately 18 000 000 elements to ensure that the major morphological features of the mandible were not obliterated in the process. The decimation steps were alternated with automatic cleaning using the ‘Mesh Doctor’ function in Geomagic Wrap to remove small tunnels, holes, open edges and sharp spikes, all of which could potentially interfere with finite element meshing. After the mandible mesh cleaning was completed, the cranium mesh was imported and digitally articulated with the mandible. The anteroposterior axis was set in the *x*-axis direction, the lateral axis in the *y*-axis direction and the dorso-ventral axis in the *z*-axis direction.

Using the digital skull mesh, we modelled (i) mandibular morphology without alveolar torus, with a small torus, and with a large torus, (ii) gape angles at 0° (jaw occlusion), 30° and 60°, and (iii) muscle adductor contractions under a default carnivoran jaw adductor scenario, an added novel torus component of the masseter originating from the area immediately ventral to the torus on the mandible and inserting in the general masseter area on the zygomatic arch, and the novel torus muscle attaching to a hypothetical new insertion site immediately posterior to the upper tooth row on the posterior margin of the maxilla (figure 1K). Alveolar tori were introduced to the base jaw model using fossil specimens as references and the ‘deform region’ tool in the ‘Sculpt’ module of Geomagic Wrap. A small-sized alveolar torus was created by centring a deformation region on the surface of the mandibular corpus immediately ventral to the m1 (carnassial) tooth; a selection diameter of 10.2 mm, ellipse shape of 2.0 and a deformation distance of 6.6 mm was applied to produce a torus similar to two observed fossil specimens of *Nimravus brachyops* (University of California Museum of Paleontology, UCMP 76603, 76111; figure 1A–F). A large torus was generated in a similar manner, but with a selection diameter of 14 mm, a deformation distance of 3.0 mm and the same ellipse shape as the small-sized torus to create a more pronounced extension from the mandibular corpus, similar to the alveolar torus observed on another fossil specimen of *N. brachyops* (UCMP 76106; figure 1G–I, electronic supplementary material, figure S1). For gape angle adjustment, the cranium mesh was rotated relative to the mandible around the temporomandibular joint to 30° or 60°, respectively. Finally, muscle insertion areas for the temporalis, masseter and lateral pterygoid muscles were highlighted on both the cranial and mandibular regions using osteological evidence (bone rugosity) and with reference to prior work [13]. For simulation scenarios incorporating a novel torus muscle, we highlighted a region ventral to the location of the alveolar torus on the mandibular corpus that is roughly the same anteroposterior extent as the length of the torus.

To generate muscle adductor forces, we used the BoneLoad script from [14], implemented in MATLAB (MathWorks, Natick, MA, USA). First, the mandibular muscles were exported in Nastran format (*.dat) using Strand7 finite element software (Strand7 Pty Ltd, Sydney, Australia). The magnitude of total force produced by each muscle was calculated to be muscle area × 0.3 N, as a proxy for force estimates calculated using physiologic cross-sectional areas [15,16]. Next, the centroids towards which each mandibular jaw adductor contracted were calculated in Geomagic Wrap on each corresponding cranial muscle insertion site using the ‘centre of gravity’ analysis tool. Both left and right jaw adductors were included in our analyses, and we further adjusted the balancing side muscle recruitment percentage to be 60% of the working side force magnitude [17]. All muscle forces were calculated using the ‘tangential’ force option in BoneLoad and exported from MATLAB as Nastran files.

The cleaned mandibular mesh models (base, small torus, large torus models) were imported into Strand7, cleaned (zipping duplicated nodes generated by the stl export step in Geomagic Wrap) and solid-meshed using four-noded tetrahedral elements. Given the similarity in the models tested in this study, we used one high-resolution solid mesh for each morphology (but see [18] for modelling recommendations for broad comparative samples). The solid mesh models were combined with individual muscle mesh models generated from BoneLoad to create the full list of model conditions tested in the study (electronic supplementary material, table S1). All models were then assigned a single set of material properties, with elastic (Young’s) modulus of 18 GPa and Poisson ratio of 0.3 [17]; plate elements loaded with muscle forces were additionally assigned a depth of 0.0001 mm in order to properly conduct the surface muscle loads from plate elements into the bony portion of the model represented by solid elements [14]. All models were constrained from translation in all directions on one of the temporomandibular joints, allowed to translate in the lateral direction on the other jaw joint and constrained from dorsoventral movement only at the tips of the paraconid and protoconid cusps of the m1 teeth, respectively. These boundary conditions simulated a bite using the shearing component of the carnassial tooth. The simulation scenarios were solved using the linear static solver in Strand7.

Four output values were collected from the simulations to evaluate performance differences among the models. (i) Stored strain energy, or the work done by the applied loads to deform the mandible model, is a measure of the overall stiffness of the structure under deforming forces [19]. (ii) Maximum von Mises strain, a measurement of the largest deformation in the model, was taken after removing the top 2% of strain values to avoid sampling inaccurate artefacts near the regions of nodal constraints. (iii) Reaction forces at the two cusp constraints were collected to represent the total simulated bite force output from each scenario. Finally, (iv) mechanical efficiency was calculated as the ratio between total reaction forces from the two carnassial cusps and the total amount of input force from all the adductor muscles modelled. Statistical differences among groups of models were assessed using non-parametric Kruskal–Wallis tests, and for differences significant at the *p* < 0.05 level, *post hoc* Dunn tests were performed to identify pairwise differences among model treatments. Model simulation results are visualized using box plot functions in the ggplot2 library in R [20].

A summary of the model parameters is listed in electronic supplementary material, table S1. All muscle configuration and jaw models are included in electronic supplementary material, data S1.

## 3. Results

**Figure 2.**
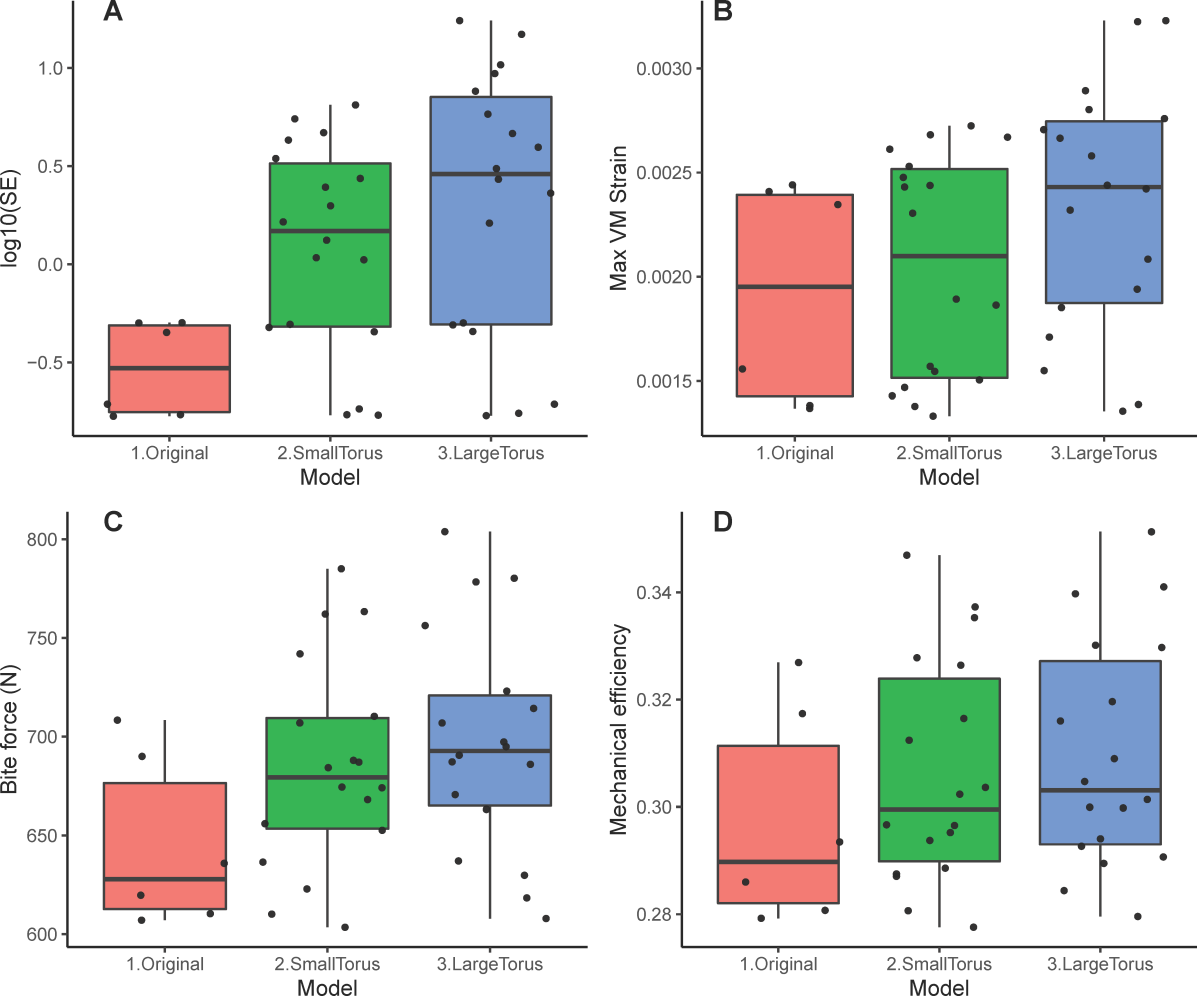
Box plots of mechanical performance traits in different jaw models. (A) Log_10_ strain stored strain energy (in Joules), (B) a maximum von Mises strain of 98%, (C) bite reaction force (in Newtons) and (D) mechanical efficiency.

Comparing all simulation scenarios across the original, small-torus and large-torus jaw models, we found that the original model has significantly lower strain energy compared with models with tori (figure 2A; electronic supplementary material, figure S2, tables S1, S2). Additionally, there is a trend of increasing values from original to small to large torus models for the other three traits (maximum von Mises strain, simulated bite force and mechanical efficiency), but those differences are not statistically significant (figure 2B,D).

**Table 1.** Kruskal–Wallis test of jaw model differences. SE, stored strain energy; VM, von Mises.

	mandible model	
	*χ*2	*p*
log_10_(SE)	7.66	0.02
VM strain	3.67	0.16
simulated bite force	3.7	0.16
mechanical efficiency	2.16	0.34

	model + gape	
	*χ*2	*p*
**0°**		
log_10_(SE)	4.55	0.1
VM strain	3.1	0.21
simulated bite force	3.08	0.21
mechanical efficiency	1.86	0.39
**30°**		
log_10_(SE)	3.35	0.19
VM Strain	0.88	0.65
simulated bite force	3.08	0.21
mechanical efficiency	2.82	0.24
**60°**		
log_10_(SE)	1.5	0.47
VM strain	0.61	0.74
simulated bite force	3.5	0.17
mechanical efficiency	3.28	0.19

	model + muscle	
	*χ*2	*p*
**no torus**		
log_10_(SE)	0.08	0.96
VM strain	0.15	0.93
simulated bite force	0.32	0.85
mechanical efficiency	0.32	0.85
**torus to masseter**		
log_10_(SE)	3.69	0.05
VM strain	2.08	0.15
simulated bite force	0.92	0.34
mechanical efficiency	0.41	0.52
**novel torus**		
log_10_(SE)	1.64	0.2
VM strain	1.64	0.2
simulated bite force	0.92	0.34
mechanical efficiency	0.41	0.52

**Figure 3.**
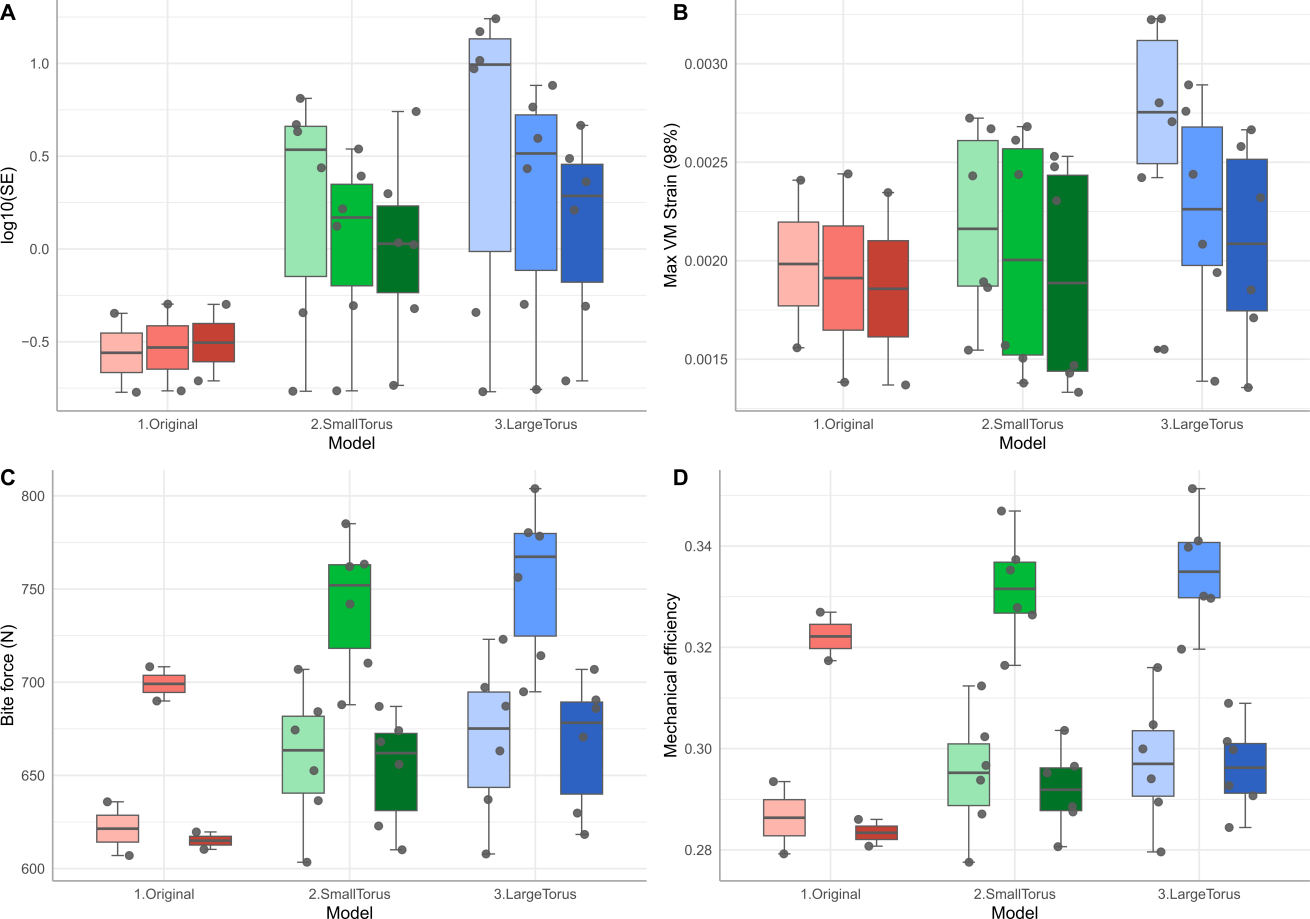
Box plots of mechanical performance traits in different jaw models, grouped by biting gape. (A) Log_10_ strain stored strain energy (in Joules), (B) a maximum von Mises strain of 98%, (C) bite reaction force (in Newtons) and (D) mechanical efficiency. Red, original mandible model; green, small torus; blue, large torus. The colour gradient for each model indicates increasing gape angle from the lighter to the darker shades.

Bite performance values do not differ significantly across the three gape angles simulated (table 1). However, torus models show a trend of decreasing strain energy and maximum von Mises strain values with higher gape angles, whereas the original model shows an increasing trend in strain energy with gape angle and a subtle decrease in strain with gape angle (figure 3A,B). In all mandible models, an intermediate gape produces the largest bite reaction force and mechanical efficiency values, whereas extreme gape angles similarly lower values within each mandible model. The original model produces the lowest forces (figure 3C,D). Strain energy and the maximum VM stress tend to decrease with gape angle (figure 3A,B), whereas simulated bite force and mechanical efficiency exhibit similar values at 0° and 60°, while a peak is observed at 30° (figure 3C,D). Simulated bite force and efficiency are significantly higher at 30° gape than at the other gapes (Kruskal–Wallis test; *p* < 0.0001, *χ*2 = 22.81 for simulation bite force and *χ*2 = 27.35 for mechanical efficiency).

The inclusion of a hypothetical novel torus muscle substantially increases jaw strain energy but less significantly increases maximum von Mises strain (electronic supplementary material, figure S2A,B). Furthermore, the shift of the cranial insertion site of the hypothetical torus muscle from the overall masseter centroid to a hypothetical anterior torus insertion site increases both bite force and mechanical efficiency compared with simulations where no torus muscle is modelled (electronic supplementary material, figure S2C,D). None of the muscle insertion scenarios is statistically significantly correlated to performance trait values (table 1).

## 4. Discussion

The development of a variably sized alveolar torus in most individuals of the sabretooth species *N. brachyops* has been observed by palaeontologists for well over a century. However, no explicit test or evaluation of the potential functional role of this structure in mastication has hitherto been performed. We constructed hypothetical jaw models with alveolar tori of different sizes to test the hypotheses that the torus structure provided a mechanical advantage to biting in *N. brachyops,* as (i) a bony buttress against strain and also (ii) a site of novel muscle attachment. Our results do not support hypothesis 1 but provide some support for hypothesis 2. Rather than buttressing the jaw, torus presence decreased the stiffness and increased the strain of the mandible under biting loads, but also increased simulated bite force and mechanical efficiency, especially at higher gapes from 30° to 60°. The modelling of novel torus muscle attachments increased both simulated bite force and efficiency. These observations suggest that the alveolar torus is associated with modifications of the biomechanical performance of the mandible in an incremental manner rather than fundamentally changing jaw mechanics.

Although the overall trend across the four performance traits measured shows that increased torus size correlates with increased strain energy, von Mises strain, bite reaction force and mechanical efficiency values, the incorporation of different muscle configurations and gape angles provides additional insights into the form–function linkage of the alveolar torus. A large torus tends to exhibit a higher gradient of decrease in strain energy and von Mises strain with increased gape, mirroring the observations made by Chatar *et al.* [1,21] on the overall craniodental system across multiple carnivoran clades that evolved sabretoothed forms. Additionally, incorporation of a hypothetical novel torus muscle also steadily increases bite reaction force and mechanical efficiency with increased torus size, but a larger torus muscle does not further increase strain energy or von Mises strain values (figure 3A,B). Considered together, these findings suggest that a larger torus at higher gape maximizes the mechanical performance benefits among the model conditions tested. The temporal trend of enlarging alveolar torus in stratigraphically younger specimens of *Nimravus* would suggest that morphological and functional specialization in this genus is consistent with improved jaw mechanical performance provided by increasing torus size and gape. One additional aspect of the jaw morphology in *Nimravus* that deserves further consideration is the combination of a well-developed torus with an unreduced coronoid process (figure 1A). The added mechanical performance benefits of the torus at high gape angles may be limited by what gape angles are possible with a high coronoid process. Future research into potential jaw element-wide form–function linkages of a torus-equipped masticatory system would further clarify the significance of the torus in modifying the *Nimravus* musculoskeletal configuration.

In spite of support for a functional role of the alveolar torus in our simulation results, it is still unclear why such performance-enhancing morphological traits did not evolve in any other lineage. The craniodental complex associated with elongate upper canines has long been considered an example of mosaic evolution, where different morphological features that contribute to the inferred function of a complex system evolved piecemeal rather than simultaneously in time [22–24]. Another example of a non-universal feature of sabretooth lineages is the degree of development of mandibular flanges; taxa such as *Barbourofelis* develop hypertrophied mandibular flanges nearly as deep as the extent of the upper canines in occlusion, whereas taxa such as *Smilodon* have much more subtle chin development but still elongate upper canines. The biomechanical implications of varying degrees of flange development across taxa have yet to be tested using methods such as FEA, but the broad taxonomic occurrence of this feature suggests a more typical convergence rather than a one-off evolutionary singularity. We speculate that in the case of the alveolar torus, the genetic basis required for consistently heritable morphology was much rarer than for the evolutionary exaggeration of other craniodental traits associated with sabretooths. This trait may have been initiated as a rare genetic mutation, which would explain why its development is not uniform across the genus, being more developed in North American than in European specimens. This would also indicate that directional evolution alone would not have been sufficient to fix or exaggerate the alveolar torus trait in a single genus, but a combination of chance genetic mutation [25] followed by incremental enlargement through natural selection would have been a plausible pathway for the evolution of the torus in *Nimravus*.

## 5. Conclusions

For nearly 150 years, a unique morphological feature of the mandible in the sabretooth *Nimravus* has been observed and commented on by multiple generations of palaeontologists. The potential function of the mandibular alveolar torus was tested for the first time here using finite element simulations, which showed that larger torus sizes are associated with a limited increase in strain in exchange for steady increases in simulated bite force and mechanical efficiency. These functional advantages are amplified at larger gape angles typical of more specialized sabretooths and indicate that alveolar torus development is linked to the evolution of the sabretooth craniodental complex. These new data underscore the mosaic nature of sabretooth evolution and the potential for relevant functional morphological traits to appear only once during the macroevolutionary record. Performance reinforcement of the craniodental trait complex, typically associated with elongate upper canine teeth, by rare traits such as the alveolar torus further highlights the non-deterministic nature of this extreme morphological specialization.

## Data Availability

All data necessary for the interpretation and verification of results are available within the article or as part of the electronic supplementary material. Supplementary material is available online [26].
